# Assessing stakeholder perceptions to guide social and ecological fit of marine protected areas

**DOI:** 10.1016/j.isci.2024.110952

**Published:** 2024-09-13

**Authors:** Victor Brun, John Roderick V. Madarcos, Anna J. Celis, Lota A. Creencia, Georgina G. Gurney, Joachim Claudet

**Affiliations:** 1 National Center for Scientific Research, PSL Université Paris, CRIOBE, CNRS-EPHE-UPVD, Maison de l’Océan, 195 rue Saint-Jacques, 75005 Paris, France; 2 Sulubaai Environmental Foundation, Taytay, Palawan 5323, Philippines; 3 College of Fisheries and Natural Sciences, Western Philippines University, Puerto Princesa, Palawan 53000, Philippines; 4 College of Arts, Society and Education, James Cook University, Townsville, QLD 4811, Australia

**Keywords:** Marine processes, Ecology, Social sciences

## Abstract

Effective social and ecological interventions that can benefit both nature and people are needed to halt the degradation of ecosystems and subsequent negative impacts on human well-being. Marine protected areas (MPAs) are commonly used to foster the sustainability of coastal social-ecological systems. However, because MPAs are often proposed and implemented by external actors, ensuring they are fit to the local social and ecological context remains a challenge. Here, we introduce a framework to identify the place-based social and ecological goals for an MPA. We use a marine conservation project in the Philippines as a case study. We assess the perceptions of local communities and decision-makers across four categories: (i) marine importance, (ii) environmental stressors, (iii) proposed management options, and (iv) MPA goals and needs. Assessing these is a way to refine marine conservation goals locally, adapt the implementation of planned interventions, and monitor their future outcomes.

## Highlights

• Marine protected areas (MPAs) can provide social and ecological benefits
• We propose a framework dedicated to identify locally relevant goals for MPAs
• This framework is applied to a case study where shared goals are identified
• Divergences in perceptions are noted and should be a focus for management

## Graphical abstract

**Figure:**
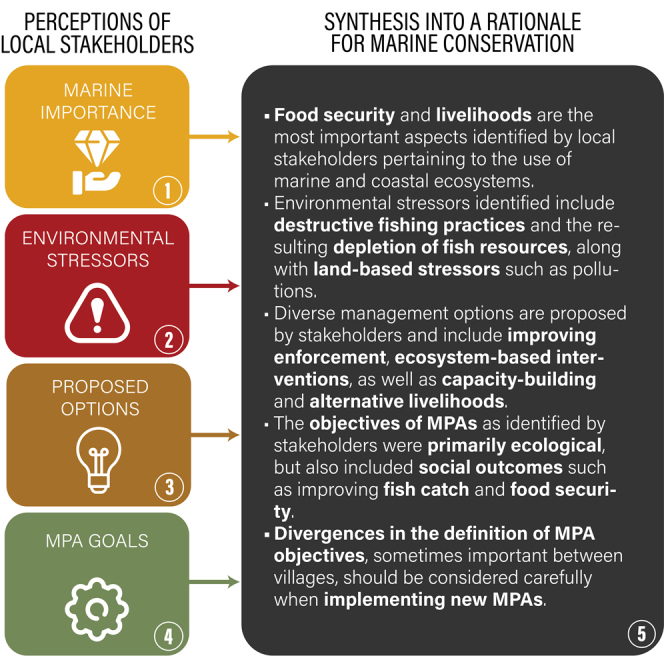

## Introduction

Coastal fishing communities can be highly dependent on marine ecosystem services, making them particularly vulnerable to marine environmental degradation or changes in access to resources.^1^^,^^2^^,^^3^^,^^4^ Improving the way coastal and marine resources are used is a great challenge as ocean-based activities such as coastal tourism, infrastructure development, and fishing are developing at an increasing pace.^5^ A variety of approaches exist to identify sustainability interventions in coastal social-ecological systems (SESs) according to their expected outcomes^6^ or local conditions.^7^ However, the identification and implementation of sustainability interventions are often driven externally,^8^ which can increase the likelihood that interventions are not fit to local contexts and fail to deliver expected positive social and ecological outcomes, or even lead to negative outcomes, including increased environmental degradation, social inequities, and conflicts.^4^^,^^9^^,^^10^^,^^11^^,^^12^^,^^13^^,^^14^^,^^15^^,^^16^^,^^17^^,^^18^ The concept of social and ecological fit represents the idea that some governance arrangements and interventions are more adequately and specifically suited to the social and ecological characteristics of the environmental problem at hand.^19^^,^^20^^,^^21^ It has been applied to study the relevance of sustainability interventions in the context of marine SESs and their ability to efficiently curb ecological threats and improve human well-being.^20^^,^^22^

Marine protected areas (MPAs), among other area-based management tools, are commonly used to improve the sustainability of coastal SESs.^23^ MPAs can deliver benefits to people across diverse well-being dimensions (Ban et al. 2019) and, thus, can be appealing to local communities.^24^ However, MPAs are also often proposed, implemented and managed by external actors, including non-governmental organizations (NGOs). While these actors can support the participation of local actors in resource governance^25^ and enhance the financial and legal capacity of MPAs,^26^^,^^27^ externally driven area-based conservation can create or exacerbate local vulnerabilities, for instance, when preventing fishers from accessing their fishing grounds.^4^^,^^14^^,^^28^ Indeed, opposition to MPAs can arise when they do not meet local needs or their benefits are oversold.^29^^,^^30^ Social and ecological fit should, therefore, be a top priority for all conservation initiatives,^20^^,^^21^^,^^22^^,^^31^^,^^32^ if not a moral obligation.^33^ As global coverage of MPAs is likely to increase at a fast pace to comply with Target 3 of the Kunming-Montréal Global Biodiversity Framework, improving the inclusivity and fit of MPAs constitutes a pillar of ocean justice and equity.^12^^,^^33^

Social assessments focusing on stakeholders’ perceptions have widely been used for MPA planning and evaluation. The perceptions of stakeholders can be informative to support multi-level governance arrangements, where the motivations and actions of different actors can be highly contrasted and need to be integrated.^34^ These can help evaluate local dependencies on marine ecosystem services,^35^ assess threats to ecosystems,^36^ help design the MPA,^37^ understand barriers and levers to a specific MPA attribute such as the level of protection,^38^ or assess local support for conservation.^39^ However, a framework designed to jointly study local actors’ perceptions on these different aspects that are of relevance to the social and ecological fit of MPAs is still lacking.

Here, based on the literature on MPA governance and effectiveness^4^^,^^10^^,^^27^^,^^39^^,^^40^^,^^41^, we develop a framework designed to understand and guide the fit of MPAs to the local social and ecological context by assessing stakeholders’ perception across four categories: (i) marine importance, (ii) environmental stressors, (iii) proposed management options, and (iv) MPA goals. Assessing the perception of marine and coastal ecosystems is a way to identify which benefits MPAs aim to improve or sustain. Similarly, these communities’ perceptions of environmental stressors are informative regarding threats expected to be curbed by MPAs. Proposed management options represent a portfolio of different interventions that can include but are not limited to MPAs. Finally, asking stakeholders about the specific goals they envision for MPAs is a way to dive deeper into what they are expected to achieve in relation to the three first categories.

In order to trial our framework, propose tentative methods for data collection and analysis, and demonstrate some of the results that can be obtained and their local relevance for management, we studied a conservation project undertaken in Palawan, Philippines, where a NGO is promoting the creation of several community-based MPAs. We interviewed local resource users, researchers, and decision-makers across the four categories of our framework and synthesized their perceptions to assess the challenges and opportunities for a local social and ecological fit of that area-based conservation project.

## Results

### A framework to assess the social and ecological fit of marine protected areas

We aimed to develop a framework designed to survey the expectations of local stakeholders and the goals for a project of new community-based MPAs to guide their social and ecological fit. The survey was designed to collect perceptions in the following four categories: (i) marine importance, (ii) environmental stressors, (iii) proposed management options, and (iv) MPA goals (Table 1). The results are intended to help ensure management is more likely to be locally fit, hence aligned with local values, needs, cultural and governance systems, which means a greater likelihood of local leadership, support, compliance, trust, and therefore social and ecological outcomes.^22^^,^^39^^,^^42^ This framework can also be informative on social and ecological interactions, which is key to understand the conditions in which management can be (in)effective.^43^ We believe the target audience of such assessment can be MPA managers, whether they are NGOs, government, or local community representatives. The following sections detail each category of the framework using existing literature, and provide examples of their use in the context of area-based marine conservation and its social and ecological fit.

**Table 1:** List of themes identified through content analysis

Category	Theme	Description
Marine importance	Livelihood	Marine ecosystems are valuable because people get their livelihoods and income from them.
	Food and nutrition	Local communities get affordable and healthy food from the sea, mostly fish but also shells, shrimps and seaweeds.
	Cultural services	Local communities value marine ecosystems for their contribution to local traditions, for religious reasons and as a responsibility for future generations.
	Other services	Other ecosystem services mentioned by respondents include coastal protection, carbon capture, and tourism.
	Intrinsic value	Respondents mentioned the intrinsic value of nature, biodiversity or particular species.
	No value identified	The respondents could not identify any important aspect and services related to marine ecosystems.
Environmental stressors	Destructive fishing practices	A threat to marine ecosystems is the use of destructive fishing practices such as cyanide, dynamite, small-meshed nets or compressors.
	Depletion of marine resources	Fishery resources have decreased, and habitats have been noticeably damaged because of the increasing number of fishers and the use of destructive practices.
	Deforestation	Respondents described issues such as deforestation for timber, slash-and-burn agriculture (*kaingin*), or mangrove cutting for charcoal (*uling*).
	Social issues	Environmental issues have root causes that can be found in social issues such as poverty, lack of education and opportunities, or demography.
	Pollutions	Pollutions such as plastic pollution, wastewater, solid waste, pesticides, and fertilizers are perceived to be stressors to terrestrial and marine ecosystems.
	Climate change and disasters	Climate change, changes in weather patterns, and disasters such as typhoons and floods are affecting ecosystems and the well-being of coastal communities.
	No issue identified	No environmental issue could be identified by the respondent.
	Land-based stressors	Some land-based stressors, such as mining, erosion, the development of infrastructure, tourism, and pearl farms, are affecting terrestrial and marine ecosystems.
	Agriculture and water	Pests are affecting plantations, and water appears to be more and more scarce.
	Marine conservation	Marine conservation, particularly MPAs, is perceived as a vulnerability factor for fishers.
Proposed management options	Legal instruments & enforcement	Coercive social interventions can be put in place, such as reinforcing patrolling and arresting offenders.
	Capacity building & alternative livelihoods	Non-coercive social interventions include the increase of education, better cooperation between stakeholders, or capacity building.
	Ecosystem-based interventions	Ecosystem-based interventions such as marine reserves and ecosystem restoration activities can be efficient in responding to environmental stressors.
	No option identified	The respondent could not identify any options for facing environmental stressors.
Marine protected areas goals	MPAs for ecological sustainability	The role of MPAs is to improve the ecological habitats of their components, including fishes, corals, shells, and functions such as nurseries.
	MPAs for local actors	MPAs are made primarily to benefit neighboring communities.
	MPAs for fisheries & food	The primary goal of MPAs is to improve the status of fisheries and the food security of coastal communities.
	MPAs as coercive instruments	MPAs are seen primarily as coercive tools, guarded areas expected to expel illegal fishers, and can be a tool to apply existing fisheries regulations.
	MPAs for external actors	MPAs are primarily made for external actors, in particular tourists and resort owners.

Each theme is composed of several individual perceptions detailed in the data provided in Supplemental items. The description is a synthesis of the perceptions of respondents as aggregated in each theme11.

#### Marine importance

The first category we propose to explore is the perceived importance of the marine and coastal environment. The diversity of ways in which communities depend on environmental features constitutes an important part of their well-being, particularly in the context of coastal communities depending on fishing.^44^ The framing of this dependence as ecosystem services or nature’s contributions to people has caused some debate^44^^,^^45^^,^^46^^,^^47^; yet these definitions hold in common the idea that disrupting ecological processes or managing ecosystems will, in turn, affect human well-being either negatively or positively. Depending on contexts, MPAs can have positive or negative social outcomes on economic and health dimensions,^10^^,^^48^^,^^49^ but also on non-material elements such as sense of place and spirituality.^50^ Assessing marine importance can help for social and ecological fit,^19^ as it is informative on where and how people value, use and hence benefit from marine ecosystems. In other words, it highlights the core values and relationships between ecosystems and human well-being in all its dimensions.^14^^,^^51^ These dependencies indeed vary between contexts and within groups and are susceptible to radical change in time.^52^^,^^53^^,^^54^^,^^55^^,^^56^ Finally, it is important to note that many of the impacts of MPAs on people are direct and not mediated by changes in ecosystem services,^15^ for example, the immediate loss of agency of local fishers linked to new fishing restrictions. These impacts can be critical motivators whether or not people support management and are important to consider when designing options to fit the social-ecological context.

#### Environmental stressors

After documenting how marine ecosystems contribute to local communities’ well-being, we propose assessing what stressors are thought to potentially affect this contribution. Assessing environmental stressors can help guide the ecological fit of MPAs, defined as an “*alignment between the spatial*, *temporal and functional characteristics of biophysical problems and institutions*”.^19^ The perception of environmental stressors in marine ecosystems varies between actors, cultures and knowledge systems.^57^^,^^58^^,^^59^^,^^60^ These stressors encompass drivers linked to climate change or various pollution sources and their impacts, as well as the root causes of these threats, namely more distal drivers such as urbanization or poverty. Using the ecological knowledge of local stakeholders is a way to ensure MPAs can effectively tackle the threats at play in that particular ecosystem. It is also a step forward in assessing their expected future outcomes. For instance, if stakeholders consider the main impact to be tackled to be a decrease in the population of a specific group of species, subsequent monitoring could focus on that group of species.

#### Proposed management options

We refer to “proposed management options” as the ideas that different stakeholders bring up in the discussion as a potential solution to curb the stressors previously identified. When an MPA is planned or already exists, asking stakeholders to discuss all potential management options is a way to verify whether and in what context they mention MPAs (e.g., as a legitimate useful tool or as an intervention with limited potential). It also allows for a better understanding of stakeholders’ perceptions of MPAs, refining of specific MPA goals, and adaptive improvement of MPA management.^61^^,^^62^ Leaving space for different stakeholders to make management propositions can be vital to increasing their support and legitimacy.^63^^,^^64^ A crucial task to address this question lies in the delimitation of who community members and stakeholders are, which is typically done based on the degree and nature of their relation to local coastal and marine ecosystems.^65^ “Stakeholders” can include actors who do not belong to the community, such as provincial government representatives in charge of managing protected areas. Defining their roles in management and their role in decision-making is important to ensure coherence in the management propositions made.

#### MPA goals

The goals of MPAs can be geared toward ecological outcomes, biodiversity outcomes, or both.^23^ They can differ between community members and external actors.^66^^,^^67^ Therefore, it is vital to make them explicit, and align them as well as possible with the underlying values of stakeholder, in the interests of achieving better outcomes from area-based management. Diving deeper into how community members perceive the goals of MPAs based on their use of marine ecosystems, allows us to identify potential issues of conflict. It can also help refine goals that were initially put forward by external actors.

#### Synthesis and rationale

The last step is to build on the four aforementioned categories, integrate these results, and build what we call a rationale for MPA management. A particular rationale, in the form of a synthetic narrative, can include present goals, future ones, resources, and actors, consider potential trade-offs and conflicts, and constitute a summarized statement on how this MPA should fit into the social and ecological context. The framework we propose is exploratory and should guide subsequent decision and conflict resolution stages. The way we propose to synthesize these results is to examine alignments, unveil a shared narrative for these goals and divergences, and push for further discussions between different actors based on their potentially conflicting visions. The destination and use of this synthesis will depend on contexts, but generally, we propose that it could be useful to MPA managers, typically in the context of co-management schemes.

### Case study: Creating a network of MPAs in Palawan, Philippines

**Figure 1:**
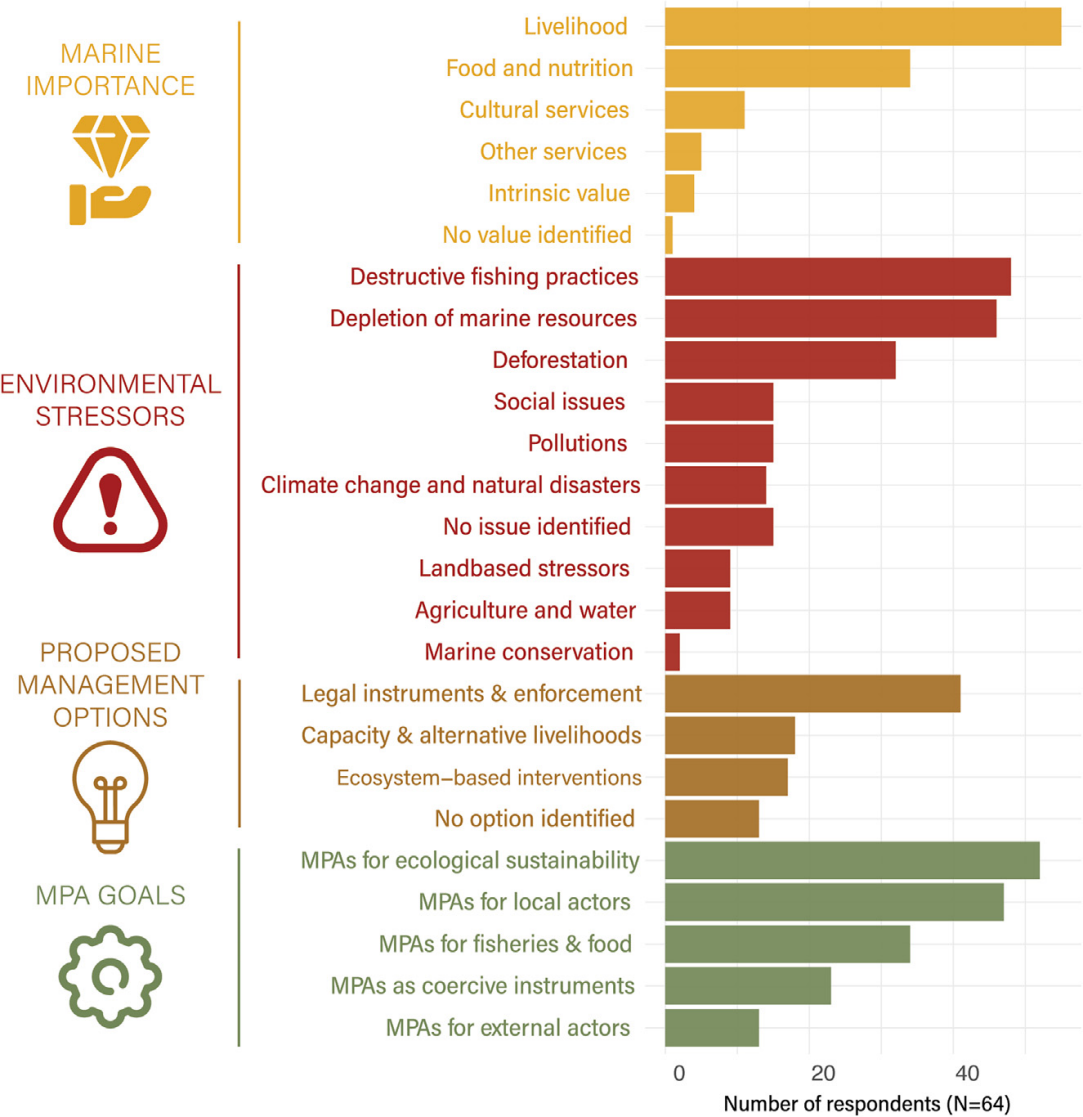
Main perceptions of different stakeholders grouped in the four perception categories proposed in our framework

Using structured interviews, we investigated the perceptions of 64 local stakeholders (53 community members and 11 decision-makers in charge of environmental management) in Palawan, Philippines, on the creation of an MPA network. After content analysis, we identified 151 individual perceptions and grouped them into 25 themes across the four categories of our framework (Table 1; Figure 1). This allowed us to study how local stakeholders define the potential needs for and goals of MPAs. We then proposed a synthesis of these perceptions and a rationale for local marine conservation projects in the form of a narrative integrating those elements.

#### Background and study site

The Philippines has a long history of community-based marine conservation.^68^^,^^69^^,^^70^^,^^71^ Most of the country’s population is coastal and depends on coral reefs and associated ecosystems for their food security and livelihoods.^72^^,^^73^ To counteract the depletion of coastal resources,^74^ public actors and NGOs have long promoted the implementation of fishery management tools.^75^ Among these, MPAs have been presented as particularly relevant, benefitting both coastal ecosystems and fishers.^76^ Such projects in the Philippines are usually initiated by NGOs and researchers in partnership with local government units and local civil society organizations.^75^

In the Shark Fin Bay locality, our case study site, there are about 7000 inhabitants spread around five coastal districts. Fishing and farming represent the main sources of livelihood. An NGO, Sulubaai Environmental Foundation (SEF), has been active in the area since 2011 and has promoted the development of marine conservation initiatives. What started as a private endeavor with the 2016 creation of the Pangatalan Island MPA managed by SEF then evolved into a project involving more local communities and decision-makers from the Municipality of Taytay, in charge of decisions related to coastal and marine ecosystems. The interviews used for this study were conducted between 2019 and 2020. The relevance of this case study to trial our framework aiming to determining locally fit goals for MPAs lies in the specific management situation in which Shark Fin Bay was at the time: SEF was starting the Sea Academy project aiming at creating new community-based MPAs and shifting from a private to a collaborative governance arrangement. At that time, the objective was to better include local communities and decision-makers in MPA co-management; hence, there was a crucial need to scrutinize the range of relevant goals for these stakeholders.

#### Perceptions of stakeholders across the four categories

##### Marine importance

The main benefits of marine ecosystems identified by respondents were provisioning ecosystem services, particularly those related to fisheries. 83% of respondents considered that the value of marine ecosystems came from their importance for local livelihoods, and 52% emphasized their importance for food security. For instance, a respondent from Depla (farmer) said: “*The very first and original way of subsistence here is the sea*, *our sea*.” The idea of subsistence is usually completely linked to the tradition of fishing, a sentiment of ownership, and generational aspects: “*The ocean is very valuable*, *especially for the next generations. If it is damaged (…)*, *they will not have a fishery and a beautiful environment anymore*” (respondent from Depla, civil servant). The most notable divergence between respondents lies in the identification of other ecosystem services, such as coastal protection, carbon sequestration, or tourism, mainly mentioned by the decision-makers from Taytay. Only one respondent (a farmer from Mabini) could not think of any value pertaining to marine ecosystems.

##### Environmental stressors

Dynamite and cyanide fishing and their effects on marine ecosystems were central in respondents’ discourses when asked about environmental stressors: 73% of them talked about destructive fishing practices, and 70% said they observed a depletion in fish resources. “*A lot of people here are using sodium [cyanide]. It kills the small fish … I think there is no other issue here*; *this is what is causing damages*” (respondent from Mabini, fisher and farmer). This perspective is shared among decision-makers: “*The main issue here is illegal fishing*: *dynamite and cyanide fishing*, *using compressors*” (respondent from Taytay, decision-maker). Respondents widely agreed on the impact of illegal practices, along with the increasing number of (legal) fishers and associated fishing efforts: fish stocks are decreasing, and it is a cause of concern for food and livelihood security. Deforestation was also an important source of discussion, in particular mangrove cutting. Many respondents noted the connectivity between ecosystems and the importance of mangrove forests as nurseries for many fish species. Some underlying social issues were described by respondents, such as poverty, increasing demography, or lack of agency. Although most respondents proposed similar descriptions of the situation, there were also some differences in interpretations. Illegal fishing, for instance, was considered to be decreasing in frequency by 9% of respondents; some also said that overfishing outweighed the effect of illegal practices. 23% of respondents could not point to any environmental stressor; some respondents explained this could be interpreted as apprehension to talk about themes such as illegal fishing and potentially pointing at other members of the community, often relatives and close friends. Some simply state they had no idea what could constitute environmental stressors in the province or the area.

##### Proposed management options

Respondents proposed a wide array of options to counteract the issues identified. We clustered them into three distinct groups (Table 1): legal instruments and enforcement, capacity building and alternative livelihoods, and ecosystem-based interventions. 20% of respondents did not identify any potential management option or considered there was none available. The most common one, identified by 62% of respondents, was to reinforce law enforcement in order to fight illegal activities: “*When it comes to illegal activities*, *there should be more law enforcement. One of the best ways is to establish MPAs*” (respondent from Taytay, decision-maker). 35% of respondents linked MPAs to more efficient law enforcement. Ecosystem-based interventions and socio-centric interventions, such as alternative livelihoods or capacity building, were more frequent among the respondents who had typically been involved in NGO and government sustainability projects: “*The solution is to give a proper livelihood to people and illegal fishers. Give them a proper job to make a living*” (respondent from Batas, fisher). Others tended to insist more on law enforcement: “*Maybe some guards could catch [illegal fishers]. The police could also arrest them*; *they could be sent to jail*” (respondent from Batas, fisher).

##### MPA goals

MPAs were most commonly perceived as a tool to increase the sustainability of marine ecosystems (79% of respondents) and fish stocks (52% of respondents) to the benefit of local communities (71% of respondents), in particular fishers. A respondent from Depla (farmer and pastor) explains: “*The purpose [of MPAs] is to offer the fish a nursery ground. And of course*, *that will produce more fish*”, and later adds that MPAs are made for “*the people of Barangay Depla*: *the fishers. But not only the fishers*, *other people too because they will eat the catch.*” Along with this discourse, many respondents pointed out that MPAs need the capacity to be efficient, in particular, legal and financial capacity: “*We have a marine sanctuary here in Mabini. But our problem is sustaining it: we cannot sustain it because the Barangay’s* *funds* *are too little*” (respondent M13, elected official). Some respondents, in particular decision-makers, pointed out the need for monitoring. Aside from this more common discourse, two divergent and more marginal definitions of MPAs should be noted. First, 3% of respondents identified MPAs as a potential threat to livelihood, such as this respondent from Silanga (elected official and fish trader): “*[Fishers] have to go far to fish now because there are protected areas.*” The example of Silanga is evocative because several nearby island resorts created private MPAs, which are now perceived by many as instruments to serve tourism rather than improve fisheries. This discourse, sometimes expressed in more neutral words, without hostility toward resort owners, was more common in Silanga, where relatively fewer people considered MPAs as dedicated to local communities. The second divergent discourse that can be noted was found in Batas and is a conception of MPAs as a way to ban fishers coming from other districts or municipalities: according to their vision, local fishers could still fish inside using a hook-and-line. A Batas (fisher) respondent explains: “*The goal of marine sanctuaries is to avoid having people from other places fishing here. For them*, *it is forbidden to fish here in our place.*” This discourse reveals the common perception that local communities own a resource despite the legal fact that marine resources are managed at a larger municipal scale and officially shared by different districts.

##### Synthesis of a rationale for conservation

Based on the discourses of local stakeholders, we established a rationale in the form of a simplified narrative summarizing the main results with the objective to guide further discussions pertaining to marine conservation in the area:

In Shark Fin Bay, food security, livelihoods, and local traditions depend on marine resources that are increasingly depleted. Destructive fishing practices, such as the use of dynamite and cyanide, and overfishing are to blame for this decline. Underlying social drivers like poverty and the lack of livelihood opportunities represent the root causes of these practices. Terrestrial drivers, including pollution and deforestation, should also be considered as a threat to marine ecosystems and well-being. Local stakeholders identified several management options to face that situation: legal instruments and coercive measures (e.g., improving legislation and patrolling), initiatives to improve capacity and shift to alternative livelihoods, and ecosystem-based interventions (e.g., MPAs). Some stakeholders cannot identify any particular option. The specific role of MPAs, in order to fit with the objectives of local resource-users, must be to preserve fisheries from illegal activities and help restore stocks, not only for local livelihoods but also for the food and nutrition security of local communities. Because some fishers perceive MPAs as dedicated to tourists and resort owners or as a potential threat to livelihood, any entity proposing the creation of an MPA should ensure the rights of local communities are respected and their voices heard.

## Discussion

Our framework helped us identify a strong overall convergence on the need for diverse and well-enforced management options. It provided concrete recommendations to foster the social and ecological fit of the MPA network project studied. In particular, it helped us conceptualize MPAs in Shark Fin Bay first and foremost as fishery management tools while highlighting their expected benefits for food security. It also showed that MPAs are locally considered by most respondents as a relevant tool, which is not the case for all contexts.^40^ This is in line with other studies conducted in the same province where environmental stressors such as overfishing and illegal fishing make local communities, along with NGOs, researchers and local governments, call for better management.^60^^,^^77^^,^^78^^,^^79^ Our framework, structured interviews, and inductive approach in the coding process allowed the discussion to be pushed further to efficiently collect the diversity of perceptions associated with marine sustainability. The rationale derived from the interviews fed the subsequent management plan produced for the Shark Fin Bay MPA network. In particular, an explicit dual goal to improve both fishing and food security was adopted for the MPA network based on our results.

As shown by Johnson et al. (2020), management interventions should be tailored to local contexts.^79^ For MPAs, this can include the size and placement of the area to be under protection or the rules on harvesting.^32^ These rules, linked to the ecological context, are also rooted in the socioeconomic context, and their reception can be different between individuals.^22^^,^^80^^,^^81^ Marine conservation navigates in very complex systems and has to arbitrate between divergent voices. In our case study, divergences in perceptions did not appear to bear a high level of potential conflict, aside probably from the idea regularly expressed in Silanga that MPAs are “made for tourists.” Conflicts between tourism and fishing have already been demonstrated in Palawan.^82^ Other case studies have shown how, generally, conflict can emerge when MPAs pay little attention to local stakes and become exclusionary to resource users.^40^^,^^82^^,^^83^ On the other hand, in places where MPAs are considered more fair, social and ecological outcomes tend to be more positive.^39^^,^^56^ Increasing participation and perception of positive outcomes often come with time.^54^ When arbitration is needed, external actors should rely on existing formal and informal decision processes to ensure both legal and equitable outcomes. Most of the differences in perceptions between stakeholder groups could be attributable to their knowledge and experience. For example, decision-makers from Taytay discussed a diversity of marine ecosystem services and MPA benefits, demonstrating a familiarity with scientific concepts, while local communities focused more on provisioning services (livelihoods and food provision in particular). There were also some disparities between different communities, as exemplified by the cases of Batas and Silanga: while most community members talked about MPAs as areas where fishing is prohibited for the benefit of local fisheries, several respondents from Batas and Silanga considered that the surrounding communities should be allowed to fish with a hook-and-line within MPAs. The presence of nearby resorts, private MPAs, and a high exogenous fishing effort in Batas can explain these differences.

Our framework proved beneficial in gaining a better understanding of the different perceptions local stakeholders had on marine sustainability and MPAs in particular. While MPAs can have a wide range of positive ecological and social outcomes,^6^^,^^10^ it clarified the specific outcomes local stakeholders were expecting in Shark Fin Bay, therefore providing locally relevant goals. Another important feature evident in our case study was how certain stakeholders’ perceptions were informative on other stakeholders’ experiences: for instance, many non-fishing farmers and decision-makers focused on the difficulties fishers were facing. The results from this research have effectively fueled the discussions on marine conservation in the study area, showing local NGOs and decision-makers that marine conservation initiatives should have fisheries and food security as a main focus along with biodiversity benefits, and should be included as a goal in subsequent management.

The support from external actors can offer important opportunities for marine conservation, ranging from increased capacity to improving the links between stakeholders, such as community-government collaborations.^26^^,^^27^^,^^84^ In Shark Fin Bay, capacity building mainly consisted of the NGO and local government unit helping local Fisherfolks Associations organize (e.g., obtaining legal recognition, organizing meetings) and providing enforcement capacity (e.g., salaries, training of guards, purchase of equipment) and monitoring capacity (e.g., scuba diving training, collaboration with citizen-science networks). In the case of small-scale fisheries management, financial or social external support should target both economic and social conditions and be maintained in time to be truly efficient.^85^ NGOs can represent a bridge between communities and governments when undertaking conservation projects and improving their social and ecological fit.^21^ In our case study, the rest of the community was involved in discussions on the planned MPAs after the initial discussions held between NGO members and community representatives. Then, the discussion was raised at the municipal level, and subsequent public hearings involving both community and government representatives were held before a formal vote for the MPAs to be legislated. Aside from potential benefits, external interventions, particularly top-down approaches, can also carry risks. In particular, MPAs have proved to be potentially exclusionary interventions, negating local users’ legal or perceived rights when improperly involving them.^10^^,^^12^

The marine conservation community at large will have to do better to settle how external actors should participate in managing marine resources, always keeping in mind the explicit goal of ensuring equitable management. Our framework can contribute to this by helping to identify local perceptions of marine importance, environmental threats, management options, and MPA goals and thus guide their social and ecological fit. While this work focuses on coastal and marine ecosystems and MPAs, its approach could be transferred to other domains, for instance, to improve the fit of terrestrial protected areas also characterized by strong social and ecological interactions^43^ or to study other area-based management tools.^86^

### Limitations of the study

Our approach also showed some limitations, mostly coming from our position as researchers who are outsiders from these communities and, for some of us, not natives of the Philippines. This might have caused certain respondents to refrain from speaking about sensitive issues such as illegal fishing. We tried to mitigate this by interviewing a wide array of respondents while insisting on the anonymity of their answers and, in practice, had many of them open up on these challenging themes. We also made sure to use vocabulary and concepts that are commonly used by these communities.

## Resource availability

### Lead contact

Further information and requests for resources should be directed to and fulfilled by the Lead Contact, Victor Brun (vbrun@ocean-climate.org).

### Materials availability

This study did not generate new unique reagents.

### Data and code availability

• Data are available in the “*data*” folder in supplemental items (Data S1).
• Code is available in the “*R*” folder in supplemental items (Data S1).
• Both data and code are also available on the first author’s GitHub (https://github.com/victor-brun/perception-sfb).

## Acknowledgments

We thank the Municipality of Taytay and the Palawan Council for Sustainable Development (PCSD) for their continuous support. We also thank the Fisherfolks Associations of Batas, Depla, Mabini, Sandoval, and Silanga for their precious help. This study was funded by the 10.13039/501100019724French Facility for Global Environment, Blancpain Ocean Commitment, 10.13039/501100011592Prince Albert II of Monaco Foundation, and the Pure Ocean Foundation. JC was supported by 10.13039/501100004431Fondation de France (MultiNet) and 10.13039/100019184BiodivERsA (METRODIVER and MOVE).

## Author contributions

V.B. and J.C. conceived the project. V.B. collected and analyzed the data. V.B., J.R.V.M., A.J.C., L.A.C., G.G.G., and J.C. identified relevant literature and participated in the development of the conceptual framework. V.B. wrote the initial manuscript, and J.R.V.M., A.J.C., L.A.C., G.G.G., and J.C. reviewed and edited it.

## Declaration of interests

The authors declare no competing interests.

## STAR★Methods

### Key resources table

**Table:** 

REAGENT or RESOURCE	SOURCE	IDENTIFIER
**Deposited data**		

Original dataset (interviews, thematic coding)	This paper	https://github.com/victor-brun/perception-sfb

**Software and algorithms**		

Rstudio version 4.3.2	R Core Team. R: A language and environment for statistical computing. R Foundation for Statistical Computing. (2020)	https://posit.co/download/rstudio-desktop/

### Experimental model and study participant details

Participants were respondents to the semi-structured interviews. Prior informed consent was obtained from all participants. The influence of gender on responses was analyzed as part of the results, and no significant difference was found through the study. 64 respondents were selected through purposive sampling, asking for recommendations from residents, representatives of Fisherfolks Associations, and different municipal offices.

### Method details

#### Sampling

64 respondents were selected through purposive sampling, asking for recommendations from residents, representatives of Fisherfolks Associations, and different municipal offices. The first group included 53 residents from five local districts spread around Shark Fin Bay: Batas, Depla, Mabini, Sandoval and Silanga, with about 10 individuals per district, men and women (58% and 42%), fishers (42%), farmers (14%), and people with other or mixed livelihoods (25%). Due to important social homogeneity in these districts and high flexibility in local livelihoods,^87^ the respondents' activities at the time of the survey did not appear to affect their perceptions, which was indicated in the important similarities in their perceptions. The second group included 11 decision-makers: five district elected officials or *barangay captains* and six decision-makers from the municipality of Taytay, representatives of the Office of the Municipal Environment and Natural Resources, Office of the Municipal Agriculturist, Office of the Municipal Tourism Development and Management. Most decisions pertaining to coastal resources management are taken at the municipal scale, making these actors central to all discussions on marine sustainability. Prior informed consent was obtained from all participants before the interview started and interviews were recorded.

#### Interview guide

Structured interviews were conducted using an interview guide (available in supplemental items) organized around seven sections: i) environmental stressors in the province, ii) ecosystem services, iii) local environmental stressors and links with fishing activities, iv) proposed management options to tackle environmental stressors, v) goals of MPAs and benefits expected, vi) perception of SEF and other NGOs, and vii) visions for the future. All respondents were confronted with the same set of initial questions, but precisions were asked to engage in deeper discussions on each topic. Interviews with community members were conducted in Filipino and with decision-makers in English. They lasted between 20 and 30 minutes.

#### Analyses

We transcribed and translated all interviews to English and performed a content analysis and thematic coding.^88^ We used an inductive approach to identify individual codes (that we termed perceptions) and classified them across the four categories of our framework (Table S1, supplemental items). We coded these perceptions in a matrix associating each respondent with the set of perceptions derived from their responses. For example, when a respondent talked about dynamite and cyanide fishing being an issue for coral reefs, the columns corresponding to “dynamite fishing” and “cyanide fishing” were checked. This coding was performed twice and checked in order to ensure its consistency. Individual perceptions were coded to be as specific as possible at first in order to allow for subsequent groupings in larger themes. For example, dynamite fishing and cyanide fishing were both grouped under the “destructive fishing practices” theme. The number of respondents for each theme was then summed to measure their relative importance in discourses. These data, along with the content of the interviews, allowed us to distinguish the dominant discourses from the more marginal perceptions and those potentially conflicting between stakeholders. Data manipulation and analyses were conducted in R Studio version 4.3.2.^89^

### Quantification and statistical analyses

This study relied on qualitative data and no statistical test was conducted. Quantitative analyses only included a measure of the frequency of themes discussed based on the thematic coding of interview transcripts conducted.

## Supplemental information

Document S1. Figure S1 and Table S1

Data S1. R code used and data analyzed to produce Figure 1
