# Supplementary material for: Assessing stakeholder perceptions to guide social and ecological fit of marine protected areas
Source: iScience. 2024 Sep 13;27(10):110952. doi: 10.1016/j.isci.2024.110952 (PMC11460489; doi:10.1016/j.isci.2024.110952)
Supplement: Document S1. Figure S1 and Table S1 [file mmc1.pdf]

iScience, Volume *27*

Supplemental information

Assessing stakeholder perceptions to guide social and ecological fit of marine protected areas

Victor Brun, John Roderick V. Madarcos, Anna J. Celis, Lota A. Creencia, Georgina G. Gurney, and Joachim Claudet

Supplementary materials – Assessing stakeholder perceptions to guide social-ecological fit of marine protected areas

Table S1. Demographics

		**Number of respondents (%) N = 64**
**Gender**	Male	37 (58%)
	Female	27 (42%)
**Residence**	Batas	13 (20%)
	Depla	13 (20%)
	Mabini	13 (20%)
	Sandoval	9 (14%)
	Silanga	10 (16%)
	Taytay	6 (9%)
**Main occupation**	Decision-maker	11 (17%)
	Fisher	27 (42%)
	Farmer	9 (14%)
	Other / no occupation	17 (27%)
**Age**	≤ 30	4 (%)
	30 < age ≤ 50	34 (53%)
	≥ 50	21 (33%)
	NA	5 (8%)

Figure S1. Interview guide

1) What are the main environmental issues here in Palawan? When & how did they start?

2) Why is marine/coastal environment important?

3) What are the main issues of fisheries here? What are the causes? When did they start?

4) What are the existing solutions and regulations, or what do you think could be a solution?

5) Do you know what an MPA is?

a) What are their goals?

b) Who are MPAs benefitting to?

c) What do MPAs need to be efficient?
