# Supplementary material for: Assessing stakeholder perceptions to guide social and ecological fit of marine protected areas
Source: iScience. 2024 Sep 13;27(10):110952. doi: 10.1016/j.isci.2024.110952 (PMC11460489; doi:10.1016/j.isci.2024.110952)
Supplement: Data S1. R code used and data analyzed to produce Figure 1 [file mmc2.zip › Data S1/R/2_Analyses.html]

2\_Analyses


# 2\_Analyses

#### Victor Brun

#### 2023-01-16

# 1. Packages and data

```
# Packages
rm(list=ls()) ; library(tidyverse) ; library(ggplot2) ; library(plyr)
library(vegan) ; library(glue)

# Data
df <-  read.csv("./../Data/general_matrix_synthesis.csv") # Matrix of perceptions grouped in themes
df_all <- read.csv("./../Data/general_matrix_allperceptions.csv") # Matrix of all ungrouped perceptions (n = 149)
```

# 2. Descriptive statistics

```
table(df$Gender) # F = 27, M = 39
```

```
## 
##  F  M 
## 27 39
```

```
table(df$Residence) # Batas = 13, Depla = 13, Mabini = 13, Puerto Princesa = 2, Sandoval = 9, Silanga = 10, Taytay = 6
```

```
## 
##           Batas           Depla          Mabini Puerto Princesa        Sandoval 
##              13              13              13               2               9 
##        Silangga          Taytay 
##              10               6
```

```
table(df$Job_1)
```

```
## 
##  Bgy Captain          BHW        Buyer       Farmer    Fisherman  Housekeeper 
##            5            1            1            9           27            5 
## Municipality     Sarisari    Scientist      Teacher 
##            6            8            2            2
```

```
table(df$Job_2)
```

```
## 
##             Businessman      Farmer   Fisherman     Kagawad      Pastor 
##          52           3           5           1           2           1 
##    Sarisari 
##           2
```

```
table(df$Involved_project) # No = 36, Yes = 30
```

```
## 
## Decision-makers and scientists                             No 
##                              8                             36 
##                            Yes 
##                             22
```

```
table(df$Knowledge_NGO) # No = 38, Yes = 28
```

```
## 
##  No Yes 
##  38  28
```

```
table(df$Knowledge_MPA) # No = 5, Yes = 61
```

```
## 
##  No Yes 
##   5  61
```

# 3. Number of respondents per perception

```
# Dataframe summary of all perceptions
df_summary <-  df %>% 
  select(13:39) %>%
  summarise_all(sum)
df_summary <-  t(df_summary) %>% data.frame
colnames(df_summary) <- "Total"
df_summary$Perception <- rownames(df_summary)

# Relevel factors
df_summary$Perception <- factor(df_summary$Perception, levels = c("value_livelihood",
                                                    "value_food",
                                                    "value_cultural",
                                                    "value_services",
                                                    "value_intrinsic",
                                                    "value_no_value",
                                                    "issue_destructive_practices",
                                                    "issue_depletion_resources",
                                                    "issue_deforestation",
                                                    "issue_social_causes",
                                                    "issue_pollution",
                                                    "issue_climate_disasters",
                                                    "issue_no",
                                                    "issue_landbased_stressors",
                                                    "issue_agriculture_water",
                                                    "issue_conservation",
                                                    "solution_sociocentric_coercive",
                                                    "solution_capacity_alternative",
                                                    "solution_ecocentric",
                                                    "solution_no",
                                                    "mpas_ecological_sustainability",
                                                    "mpas_for_locals",
                                                    "mpas_need_capacity",
                                                    "mpas_fishery",
                                                    "mpas_coercive",
                                                    "mpas_need_support",
                                                    "mpas_for_external"))
df_summary$Perception <- fct_rev(df_summary$Perception)

# Add theme
df_summary <- df_summary %>%
  mutate(Theme = case_when(str_starts(as.character(Perception), "value") ~ "Environmental well-being",
                           str_starts(as.character(Perception), "issue") ~ "Environmental stressors",
                           str_starts(as.character(Perception), "solution") ~ "Possible options",
                           str_starts(as.character(Perception), "mpas") ~ "Perception of MPAs"
                           ))
df_summary$Theme <- factor(df_summary$Theme, levels = c("Environmental well-being", "Environmental stressors", "Possible options", "Perception of MPAs")) #Reorder theme factors

# Recode perception factors
df_summary$Perception <- fct_recode(df_summary$Perception,
                     "Livelihood" = "value_livelihood",
                     "Food and nutrition" = "value_food",
                     "Cultural services" = "value_cultural",
                     "Other services" = "value_services",
                     "Intrinsic value" = "value_intrinsic",
                     "No value identified" = "value_no_value",
                     "Destructive fishing practices" = "issue_destructive_practices",
                     "Depletion of marine resources" = "issue_depletion_resources",
                     "Deforestation" = "issue_deforestation",
                     "Social issues" = "issue_social_causes",
                     "Pollutions" = "issue_pollution",
                     "Climate change and natural disasters" = "issue_climate_disasters",
                     "No issue identified" = "issue_no",
                     "Landbased stressors" = "issue_landbased_stressors",
                     "Agriculture and water" = "issue_agriculture_water",
                     "Marine conservation" = "issue_conservation",
                     "Legal instruments & enforcement" = "solution_sociocentric_coercive",
                     "Capacity & alternative livelihoods" = "solution_capacity_alternative",
                     "Ecosystem-based interventions" = "solution_ecocentric",
                     "No option identified" = "solution_no",
                     "MPAs for ecological sustainability" = "mpas_ecological_sustainability",
                     "MPAs for local actors" = "mpas_for_locals",
                     "MPAs for fisheries & food" = "mpas_fishery",
                     "MPAs as coercive instruments" = "mpas_coercive",
                     "MPAs for external actors" = "mpas_for_external",
                     "MPAs need capacity" = "mpas_need_capacity",
                     "MPAs need compliance & support" = "mpas_need_support",
)

# Barplot
ggplot(df_summary) +
  aes(x = Perception, y = Total, fill = Theme) +
  geom_col(position = "dodge", alpha=.9) +
  labs(x = "", y = "Number of respondents", fill = "Category of perceptions") +
  theme_minimal() +
  scale_fill_manual(values = c("#E2A525", "#A51E23", "#A56F29", "#728857")) +
  coord_flip()
```

# 4. Number of respondents per perception depending on NGO knowledge

```
# Creating "df2" summing all the answers per group
df2 = df %>% 
  group_by(Knowledge_NGO) %>%
  select(13:39) %>%
  summarise_all(sum) %>%
  t() %>%
  data.frame
```

```
## Adding missing grouping variables: `Knowledge_NGO`
```

```
colnames(df2) = c("No previous knowledge of the NGO", "Previous knowledge of the NGO")
df2 <-  df2[-1,] %>%
  mutate_all(as.numeric)
df2[,1] <- df2[,1]/38
df2[,2] <- df2[,2]/28

# Relevel factors
df2$Perception = rownames(df2)
df2$Perception <- factor(df2$Perception, levels = c("value_livelihood",
                                                    "value_food",
                                                    "value_cultural",
                                                    "value_services",
                                                    "value_intrinsic",
                                                    "value_no_value",
                                                    "issue_destructive_practices",
                                                    "issue_depletion_resources",
                                                    "issue_deforestation",
                                                    "issue_social_causes",
                                                    "issue_pollution",
                                                    "issue_climate_disasters",
                                                    "issue_no",
                                                    "issue_landbased_stressors",
                                                    "issue_agriculture_water",
                                                    "issue_conservation",
                                                    "solution_sociocentric_coercive",
                                                    "solution_capacity_alternative",
                                                    "solution_ecocentric",
                                                    "solution_no",
                                                    "mpas_ecological_sustainability",
                                                    "mpas_for_locals",
                                                    "mpas_need_capacity",
                                                    "mpas_fishery",
                                                    "mpas_coercive",
                                                    "mpas_need_support",
                                                    "mpas_for_external"))
df2$Perception <- fct_rev(df2$Perception)

# Recoding factors
df2$Perception <- fct_recode(df2$Perception,
                     "Livelihood" = "value_livelihood",
                     "Food and nutrition" = "value_food",
                     "Cultural services" = "value_cultural",
                     "Other services" = "value_services",
                     "Intrinsic value" = "value_intrinsic",
                     "No value identified" = "value_no_value",
                     "Destructive fishing practices" = "issue_destructive_practices",
                     "Depletion of marine resources" = "issue_depletion_resources",
                     "Deforestation" = "issue_deforestation",
                     "Social issues" = "issue_social_causes",
                     "Pollutions" = "issue_pollution",
                     "Climate change and natural disasters" = "issue_climate_disasters",
                     "No issue identified" = "issue_no",
                     "Landbased stressors" = "issue_landbased_stressors",
                     "Agriculture and water" = "issue_agriculture_water",
                     "Marine conservation" = "issue_conservation",
                     "Legal instruments & enforcement" = "solution_sociocentric_coercive",
                     "Capacity & alternative livelihoods" = "solution_capacity_alternative",
                     "Ecosystem-based interventions" = "solution_ecocentric",
                     "No option identified" = "solution_no",
                     "MPAs for ecological sustainability" = "mpas_ecological_sustainability",
                     "MPAs for local actors" = "mpas_for_locals",
                     "MPAs for fisheries & food" = "mpas_fishery",
                     "MPAs as coercive instruments" = "mpas_coercive",
                     "MPAs for external actors" = "mpas_for_external",
                     "MPAs need capacity" = "mpas_need_capacity",
                     "MPAs need compliance & support" = "mpas_need_support",
)

# Representing all proportions using a barplot
df3 = pivot_longer(df2, c(`No previous knowledge of the NGO`, `Previous knowledge of the NGO`))
barplot = ggplot(df3) +
  aes(x = Perception, y = value) +
  geom_col(aes(fill = name), position = "dodge") +
  labs(x = "", y = "% of respondents that identified this theme", fill = "Group of respondents") +
  scale_fill_manual(values = c("black", "light grey")) +
  theme_minimal()
barplot + coord_flip()
```

```
rm(df2,df3)
```

# 5. Statistical analyses

```
# Computing total number of perceptions per respondent
df <- df %>%
  mutate(N_Perceptions = rowSums(.[13:37]))

# Computing PCOA
rownames(df) <- df$ID
dist <- vegdist(df[13:37])
pcoa <- cmdscale(dist, eig=T, add=T)
positions <- pcoa$points
colnames(positions) <- c("pcoa1", "pcoa2")

# Creating groups
Fisher <- ifelse(df$Fisher == "Yes", "Fisher", "Non fisher")
Knowledge_NGO <- ifelse(df$Knowledge_NGO == "Yes", "Knowledge NGO", "No Knowledge NGO")
Knowledge_MPA <- ifelse(df$Knowledge_MPA == "Yes", "Knowledge MPA", "No Knowledge MPA")
Involved_project <- case_when(df$Involved_project == "Yes" ~ "Involved in NGO projects", 
                              df$Involved_project == "No" ~ "Not involved in NGO projects",
                              TRUE ~ "Decision-makers and scientists")
Gender <- df$Gender

# Percentage of variation explained
percent_explained <- format(round(100*pcoa$eig/sum(pcoa$eig), digits=1), nsmall=1, trim=T ) # Compute
labs <- c(glue("PCo 1 ({percent_explained[1]}%)"), 
          glue("PCo 2 ({percent_explained[2]}%)")) # Create labs for plot

# Plot PCoA
positions %>%
  as_tibble(rownames = "samples") %>%
  ggplot(aes(x=pcoa1, y=pcoa2, col = Involved_project, shape = Fisher, size = df$N_Perceptions)) +
  geom_point(alpha=.7) +
  labs(x = labs[1], y = labs[2], shape = "Livelihood of respondent", 
       color = "Profile of respondent", size = "Diversity of perceptions") +
  scale_color_brewer(palette = "Set2") +
  theme_minimal()
```

```
# PERMANOVA
perm <- adonis2(df[13:37] ~ Knowledge_NGO + Knowledge_MPA + Involved_project + Gender + Residence + Fisher, data=df, permutations = 999, method = "bray")
perm
```

```
## Permutation test for adonis under reduced model
## Terms added sequentially (first to last)
## Permutation: free
## Number of permutations: 999
## 
## adonis2(formula = df[13:37] ~ Knowledge_NGO + Knowledge_MPA + Involved_project + Gender + Residence + Fisher, data = df, permutations = 999, method = "bray")
##                  Df SumOfSqs      R2       F Pr(>F)    
## Knowledge_NGO     1   0.6442 0.07640  7.5553  0.001 ***
## Knowledge_MPA     1   0.9929 0.11776 11.6445  0.001 ***
## Involved_project  2   0.5707 0.06768  3.3465  0.003 ** 
## Gender            1   0.1798 0.02132  2.1083  0.074 .  
## Residence         4   1.1485 0.13621  3.3674  0.001 ***
## Fisher            1   0.2060 0.02443  2.4156  0.042 *  
## Residual         55   4.6895 0.55619                   
## Total            65   8.4314 1.00000                   
## ---
## Signif. codes:  0 '***' 0.001 '**' 0.01 '*' 0.05 '.' 0.1 ' ' 1
```

```
# RDA
rda <- rda(df[13:37], data = df, distance = "bray")
plot(rda)
```

```
# ANOVA on diversity of perceptions
aov <- anova(lm(N_Perceptions ~ Knowledge_NGO + Knowledge_MPA + Involved_project + Gender + Residence + Fisher, data=df))
aov
```

```
## Analysis of Variance Table
## 
## Response: N_Perceptions
##                  Df  Sum Sq Mean Sq F value    Pr(>F)    
## Knowledge_NGO     1   6.054   6.054  2.2741    0.1373    
## Knowledge_MPA     1  76.901  76.901 28.8891 1.606e-06 ***
## Involved_project  2  92.221  46.111 17.3222 1.464e-06 ***
## Gender            1   0.149   0.149  0.0560    0.8139    
## Residence         4  12.399   3.100  1.1644    0.3365    
## Fisher            1   2.490   2.490  0.9354    0.3377    
## Residuals        55 146.407   2.662                      
## ---
## Signif. codes:  0 '***' 0.001 '**' 0.01 '*' 0.05 '.' 0.1 ' ' 1
```
